# YAP/TAZ-Hippo pathway mediates the tumorigenesis of various cancers through post-translational modification represented by ubiquitination

**DOI:** 10.1038/s41420-025-02319-w

**Published:** 2025-02-03

**Authors:** Fangshi Xu, Zongyu Li, Hao Guan, Jiancang Ma

**Affiliations:** Department of Vascular Surgery, The Second Affiliated Hospital of Xi’an Jiaotong University, Xi’an, Shaanxi Province China

**Keywords:** Oncogenes, Tumour biomarkers

## Introduction

The Hippo signalling pathway, an evolutionarily conserved mechanism, plays a crucial role in developmental biology, including organ growth, development, embryogenesis, and tissue regeneration [1]. Given its central role in regulating cell proliferation and differentiation, its involvement in tumorigenesis is a logical extension. Substantial evidence highlights the pathway’s critical contributions to cancer invasion, progression, immune evasion, and therapeutic resistance [2]. As a key regulatory axis in human cancers, the Yes-associated protein (YAP)/TAZ-Hippo pathway influences the development and outcomes of various malignancies. In breast cancer (BC), hyperactivation of TAZ drives luminal cells toward a basal phenotype, leading to the onset of basal-like BC, the most aggressive subtype of the disease [3]. In glioblastoma (GBM), the Hippo/TAZ axis promotes angiogenesis through the release of vascular endothelial growth factor C [4]. Similarly, in head and neck squamous cell carcinoma (HNSCC), TAZ enhances cancer stemness by activating the transcription factor SOX2 [5]. These findings underscore the YAP/TAZ-Hippo axis as a pan-cancer driver.

A recent study by Zhang et al. demonstrated that USP12 facilitates gastric cancer (GC) progression by stabilising YAP [6], revealing potential therapeutic targets within the Hippo pathway. These insights suggest significant opportunities for targeting this pathway in cancer treatment. Here, we critically review the core mechanisms of the Hippo pathway in cancer initiation and progression, with a particular focus on post-translational modifications (PTMs),such as ubiquitination. This review aims to outline the regulatory landscape of the Hippo pathway in human cancers and propose directions for future research.

## Activation of YAP/TAZ drives malignant progression of various cancers

The Hippo signalling pathway operated through a classical kinase cascade involving sterile 20-like kinase 1/2 (MST1/2) and large tumour suppressor 1/2 (LATS1/2) [7]. Mechanistically, MST1/2 phosphorylates and activates LAST1/2, which in turn phosphorylates the downstream effectors YAP and transcriptional coactivator with PDZ-binding motif (TAZ). Phosphorylated YAP and TAZ are sequestered in the cytoplasm and subsequently inactivated via proteasome-mediated degradation. As transcriptional cofactors, YAP and TAZ interact with various transcription factors, particularly TEA DNA-binding proteins (TEADs). This interaction drives cell proliferation and survival by regulating downstream effector genes such as CCN2, BIRC5 and AXL, thereby establishing the YAP/TAZ-TEAD axis as the central mechanism of Hippo pathway-mediated cancer regulation [8].

The oncogenic role of USP12 aligns with this regulatory framework, as demonstrated by Zhang et al. [6]. USP12 co-localises with YAP in both the cytoplasm and nucleus and enhances YAP expression by inhibiting K48-linked polyubiquitination at the YAP K315 site. Furthermore, USP12 upregulates the classical YAP target genes CTGF and TEAD, consistent with its role in stabilising YAP expression. These findings highlight how dysregulation of YAP/TAZ and their associated regulatory components profoundly influences tumorigenesis.

## An emerging bridge spanning ubiquitination to the Hippo pathway

Ubiquitination, a critical PTM, is regulated by a cascade of enzymes, including ubiquitin-activating enzymes (E1), ubiquitin-conjugating enzymes (E2), ubiquitin ligases (E3) and deubiquitinases (DUBs) [9]. Typically, polyubiquitinated proteins are targeted for degradation via the proteasome, thereby influencing the localisation and stability of substrate proteins. Increasing research has established strong connections between ubiquitination and the Hippo signalling pathway. Similar to USP12 [6], the DUB OUTB1 has been shown to deubiquitinate YAP at multiple lysine sites, thereby inhibiting its degradation [10]. Other ubiquitin-modifying enzymes also play pivotal roles in YAP-mediated cancer progression. For example, the E3 ligase HERC3 disrupts YAP/TAZ degradation by binding to β-TrCP, effectively preventing ubiquitination-induced proteolysis [11]. Interestingly, while certain E3 ligases inhibit YAP/TAZ degradation, others promote the ubiquitination and degradation of core Hippo pathway regulators to facilitate cancer progression. For instance, WWP2 accelerates LATS1 ubiquitination and subsequent degradation, driving GC development [12].

Advancements in this field have led to the development of molecular inhibitors targeting components of the ubiquitin-proteasome system (UPS), offering significant therapeutic potential [9]. Notably, a groundbreaking study published in *Cell* identified the E3 ligase Cop1 as a critical target for cancer immunotherapy [13]. This topic will be explored further in subsequent sections. In summary, the interplay between ubiquitination and the YAP/TAZ-Hippo axis provides promising opportunities for novel cancer therapies.

## Targeting UPS: Emerging therapeutic options

The UPS, comprising E1, E2, E3 enzymes and DUBs, is crucial for maintaining protein quality control and homeostasis, playing a significant role in cancer progression. Numerous molecular inhibitors targeting various UPS components have demonstrated substantial therapeutic potential. For instance, Pevonedistat (MLN4924), a selective inhibitor of the E1 regulatory subunit, has shown promise in inducing cancer cell death and has been evaluated in several clinical trials [14]. For instance, in patients with metastatic non-small-cell lung cancer (NSCLC), pevonedistat achieved a median progression-free survival (PFS) of 4.1 months and an objective response rate (ORR) of 22% [15]. However, no E2 enzyme inhibitors have yet reached clinical trials, with candidates like NSC697923 and CC0651 remaining at the experimental stage [14]. E3 ligase inhibitors have garnered particular attention due to their pivotal role in ubiquitin conjugation and their high substrate specificity [9]. In a phase I clinical trial (NCT02016729), the MDM2 inhibitor AMG 232 achieved a 20% remission rate in patients with refractory acute myeloid leukaemia (AML) [16]. Similarly, DUB inhibitors show significant potential because of their high substrate specificity and catalytic properties [17]. For example, mitoxantrone, a USP11 inhibitor, achieved a disease control rate of up to 50% in patients with advanced breast cancer [15]. In addition to cancer therapy, DUB inhibitors have applications in treating other diseases. During research on severe acute respiratory syndrome, 6-mercaptopurine (6-MP) was identified as a specific inhibitor of papain-like protease (PLpro), a cysteine protease with deubiquitinating activity [18]. Structural comparisons suggest that 6-MP may also inhibit USP14, further broadening its therapeutic potential [19]. Currently, 6-MP is widely used in cancer treatment; in a phase II clinical trial, patients with BRCA mutations achieved a clinical benefit rate of 33% following 6-MP therapy [20]. These findings underscore the potential of targeting DUBs for cancer treatment.

Among UPS-related targets, proteasome inhibitors have achieved the most successful clinical applications [9]. As the effector phase of ubiquitination regulation, proteasome inhibition prevents the degradation of tumour suppressor proteins, thereby suppressing malignant progression [21]. For example, carfilzomib and oprozomib, two classical proteasome inhibitors, induce apoptosis in HNSCC by upregulating the pro-apoptotic gene Bik [22]. Several FDA-approved drugs, including carfilzomib, bortezomib, oprozomib, and delanzomib, target proteasome activity. In multiple myeloma (MM), carfilzomib achieved an ORR of 87.1% and a median PFS of 26.3 months [23]. In summary, targeting the UPS offers a promising strategy for expanding the anti-cancer arsenal and improving patient outcomes.

## Post-transcriptional modification mediates cancer progression via regulating Hippo activities

**Fig. 1:**
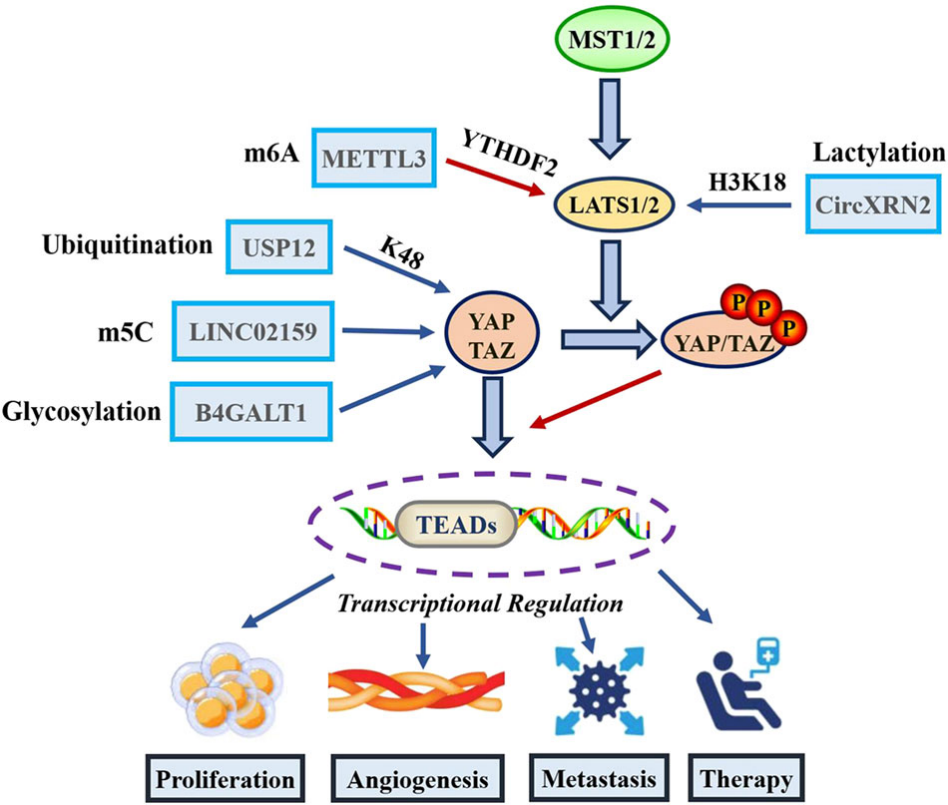
Multiple post-transcriptional modifications regulate cancer progression via manipulating the activity of the Hippo signalling pathway. MST1/2, LATS1/2 and YAP/TAZ are the core regulatory components of the Hippo signalling pathway. Upon activation of the Hippo pathway, LAST1/2 promotes YAP/TAZ phosphorylation, inactivating them. YAP/TAZ act as transcriptional activators and, together with TEADs (a pivotal family of transcription factors), promote the transcriptional process of multiple downstream target genes, thereby affecting various tumour biological processes. The blue arrow indicates that the transcription of the target gene is upregulated through specific post-transcriptional modifications (PTMs), whereas the red arrow represents transcriptional repression.

Various PTM mechanisms play crucial roles in cancer progression by modulating Hippo pathway activity (Fig. 1). Among these, N6-methyladenosine (m6A) is the most prevalent and evolutionarily conserved PTM in eukaryotic RNA. Typically, m6A modifications reduce RNA transcription efficiency and stability [16]. For instance, Xu Y et al. reported that the m6A writer METTL3 and reader YTHDF2 cooperatively facilitated the m6A methylation of LATS1, reducing its mRNA stability and promoting BC tumorigenesis[17]. 5-methylcytosine (m5C) is another common RNA modification with profound effects on RNA stability, protein synthesis, and post-transcriptional regulation [24]. In NSCLC, the long non-coding RNA LINC02159 promotes cancer progression by enhancing the m5C-mediated stability of YAP mRNA [18]. Glycosylation has emerged as a critical topic in cancer research. Glycation products, including glycan moieties, glycoproteins, and glycolipids, add layers of dynamic regulation to intracellular and intercellular signalling [25]. A recent study revealed that B4GALT1 stabilises the TAZ protein through glycosylation, thereby promoting immune evasion in lung adenocarcinoma (LUAD) by preventing PD-L1 degradation [19]. Epigenetic modifications such as histone lactylation also play vital roles in cancer regulation. Lactylation of histone lysine residues, associated with processes like angiogenesis and cell proliferation, can directly stimulate gene transcription from chromatin [20]. In bladder cancer, CircXRN2 prevents LATS1 degradation via H3K18 lactylation, thereby suppressing tumour progression by activating the Hippo signalling pathway [21]. Collectively, these findings highlight the essential role of PTMs in the regulatory landscape of YAP/TAZ-mediated cancer progression.

## Some inspirations and limitations of Zhang P’s study

The study by Zhang P et al. offers valuable insights into the ubiquitination mechanisms in cancer progression. Zhang P et al. employed a robust siRNA library screening approach targeting DUBs. This library encompassing 98 common human DUBs, utilised CTGF, a critical downstream target of YAP, as a measure of pathway activity. This strategy ensured comprehensive coverage and high accuracy in identifying USP12 as a key player in GC progression. Furthermore, immunofluorescence experiments demonstrated co-localisation of USP12 and YAP. Co-IP assays also confirmed their binding interaction. Using MG132 and CHX analyses, the study revealed that USP12’s effect on YAP protein stability depended on the proteasome pathway. This layered analytic approach effectively elucidated the specific mechanisms of ubiquitination in cancer regulation.

While this study has many strengths, several limitations should also be acknowledged. First, Zhang P et al. demonstrated that USP12 enhances the proliferation, migration and invasion of GC cells by stabilising YAP. However, YAP, as a transcriptional cofactor, facilitates the activity of TFs associated with cell proliferation. This specific downstream target gene responsible for USP12-induced GC malignancy remains unidentified. Second, this study identified USP12-mediated YAP stabilisation through K48-linked polyubiquitination (K48), excluding K63-linked polyubiquitination. However, ubiquitination-mediated protein degradation can also occur via other linkage types, such as K11-linked, K27-linked, K19-linked and M1-linked polyubiquitinations [22]. Whether USP12 influences these ubiquitination modifications remains ambiguous, leaving gaps in understanding its broader regulatory impact.

## Future perspectives

Given the critical role of ubiquitination in cancer regulation, several key research directions warrant exploration to advance this field. First, ubiquitination plays a central role in regulating cellular signalling pathways, with DUBs implicated in nearly all cancer hallmarks [26]. Targeting DUBs holds significant therapeutic promise due to their high substrate specificity [27]. However, the current generation of DUB inhibitors has faced challenges in clinical applications. For example, the novel DUB inhibitor VLX1570 demonstrated severe pulmonary toxicity in a phase I clinical trial for refractory MM, with 2 out of 14 patients (14.3%) experiencing severe respiratory failure, one of whom died [28]. These findings underscore the need for further optimisation in DUB inhibitor development. Emerging technologies, such as molecular docking and cryo-electron microscopy, may accelerate this process by improving inhibitor design and specificity [29].

Second, the diverse substrates for ubiquitination, including lysine (Lys), serine (Ser) and threonine (Thr) residues in proteins, as well as hydroxy and amino groups in lipids [30], suggest its involvement in nearly all cellular processes. This highlights the potential for synergistic or antagonistic interactions with other PTMs. For instance, OIP5-AS1, modified by N6-methyladenosine (m6A), promotes GC tumorigenesis and metastasis by suppressing Trim21-mediated hnRNPA1 ubiquitination [31]. Investigating these interrelationships can deepen our understanding of cancer pathogenesis and unveil novel regulatory mechanisms.

Third, advances in high-throughput and mass spectrometry technologies enable detailed mapping of ubiquitination landscapes across various cancer types. These profiles can aid in identifying novel biomarkers and therapeutic targets. For example, El Kaoutari A et al. used ubiquitin-dependent proteomics to pinpoint specific ubiquitination sites associated with prognosis and chemotherapy responses (e.g., to gemcitabine, oxaliplatin, 5-FU and irinotecan) in pancreatic ductal adenocarcinoma [25]. Such research has the potential to drive the development of individualised cancer management strategies, enhancing treatment efficacy and patient outcomes.

## Conclusions

Current evidence underscores the pivotal role of ubiquitination in cancer regulation via the YAP/TAZ-Hippo axis. Targeting the UPS holds significant promise for anti-cancer therapy, with proteasome inhibitors already achieving notable clinical success across multiple cancer types. Beyond ubiquitination, other PTMs, such as N6-methyladenosine (m6A) and lactylation, also contribute to cancer progression by modulating Hippo signalling activity. Importantly, these PTMs exhibit synergistic or antagonistic interactions with ubiquitination, forming a complex, multi-layered regulatory network in cancer biology. Understanding these intricate connections will provide valuable insights for designing targeted therapeutic strategies and guiding future research in this field.
